# Editorials

**Published:** 1912-01

**Authors:** 


### EDITORIALS

The Business Office of the *Journal-Record* is 1 1-2 to 5 1-2 South Broad Street.
The Editorial Office is 1014-15 Century Building.
Address all Business Communications to *Journal-Record of Medicine*, 1 1-2 to 5 1-2 South Broad Street.
Make remittances payable *to The Journal-Record of Medicine.*
On matters pertaining to the Editorial and Original Communications, address Edgar G. Ballenger, M. D., Atlanta, Ga.
Reprints of Original Articles will be furnished at cost price. Requests for the same should always be made in THE MANUSCRIPT.
We will present, postpaid, on request, to each contributor of an original article, twenty (20) marked copies of *The Journal-Record of Medicine* containing such article.

### A PETITION TO RESTORE THE CANTEEN.

Below in this issue we publish a letter to the medical profession of America from Dr. W. W. Keen of Philadelphia, in which he presents good reasons why the canteen should be restored. Two facts stand out prominently: First. Intemperance has not decreased in the army since the abolition of the canteen. Second. The increase in venereal disease has greatly increased. Therefore, it seems timely for the reconsideration of the law which forbids the sale of beer in Army posts. Physicians know better than any other class of men the far-reaching and widespread angers that following in the wake of gonorrhoea and syphilis. This danger, unfortunately, is not limited to the guilty parties, for many innocent women and babies also become victims. The efficiency of the army is probably lessened more by the disabilities produced by these disease than any other causes. With these admitted facts before us and the evident failure of the present plan, we are forced to admit the logic in Dr. Keen's reasoning and thus favor the restoration of the canteen.

*To the Medical Profession of America*:

In the Journal of the American Medical Association, December 16, was published in full (but, of course, without the 275

signatures of leading medical men all over the country) a petition to Congress to pass the Bartholdt bill restoring the canteen in the Army. The petition explains fully what the canteen really is.

My own position in the matter is this: I never place wine on my table on any social occasions whatever; I did not do so even at the weddings of my daughters and I never partake of it at any dinner or on other social occasions. If I had autocratic sway in America, I should nail up the doors of every saloon and put on them a placard "To Rent for some decent business;" and yet I am in favor of allowing beer (and be it noted that beer only would be provided in the canteen) to be sold to the soldiers. As a surgeon, I am bitterly opposed to mutilating the body of any fellowman by amputating a leg, but when I have met with the aternative that the man must either lose his leg or lose his life, I have unhesitatingly sacrificed the leg in order to save the life.

It is by a precisely similar course of reasoning that I have reached the conclusion that the canteen should be restored. As I have read over for some years the reports of the Surgeon-General of the Army I have been deeply stirred by the recent enormous increase in the prevalence of venereal diseases to nearly 20 per cent. of the army annually. In my personal relations as a surgeon, I have known no less than five of my warm professional friends who have suffered from an innocent syphilitic infection which has destroyed their health or their lives. My old friend. the late Dr. Nevins Hyde of Chicago, when I was discussing the matter with him one day told me that he had at that moment seven physicians under his care for an accidental syphilitic infection, and he mentioned several cases of physicians of his acquaintance who had committed suicide as a result of such an infection.

The wives and children of our soldiers are much more subject to accidental and innocent infection than physicians. Many of their wives are inevitably infected either with gonorrhoea or syphilis and their lives made miserable and the community deprived of healthy child-bearing women. Many of their children are doomed to blindness from gonorrhoeal infection in the very act of birth, many of them are rendered hopeless and wretched subjects of inherited syphilis; and in turn both father, mother and children may infect others.

When I read in the North American Review of March, 1911, an article by the Hon. James H. Blount, summarizing and collecting much of this information, I was not content simply to raise my eyes to Heaven and piously sigh "What a dreadful state of affairs!" and go to sleep; but I asked myself at once, "What is *my* duty as a citizen, a surgeon, and a Christian toward the men in the Army, toward their wives and children, and toward the community?"

After thinking the matter over very carefully and again referring to the Surgeon-General's Reports, I concluded that there was laid on me a duty to try to influence Congress to repeal the bill prohibiting the sale of beer in the Army posts and that the most powerful influence I could bring on Congress would be a petition from medical men alone and based on medical grounds. Accordingly I drew up the petition that is published in the Journal and sent it to a member of medical men all over the country.

I selected them from lists of our various national societies as men of unusual prominence and influence in their communities. I sent them a copy of the petition with a request to read it carefully and, if they approved of it, to sign it. A number, of course, neglected it entirely, some, I happen to know, from absence, illness and other causes.

I have received 278 signatures, three too late to be included in the petition. I have received only three refusals to sign: one because of want of information, a second because that correspondent's brother had died as a result of dissipation, the last because the third correspondent was opposed *in toto* even to the use beer, no matter what the consequences in other respects might be.

My attention has been called to the fact that this paragraph may produce the erroneous impression in the mind of some readers that not many more than 281 petitions were sent out, of which only three were returned with a refusal to sign. I, therefore, went over the list of those to whom the petition was sent and found that 484 had been sent out, 281 were returned as mentioned in the letter, leaving 203 from whom no word was received. These non-signers should be divided into four classes:

1. Those who approve of the petition but did not wish to be drawn into the controversy or to be annoyed, attacked, and even ostracized, especially in communities where the local anti-canteen spirit was very pronounced. Judging by the results of the known returns (278 to 3), it is my opinion that these may be set down as over one-half of the 203.

2. Those who did not approve of the petition but also preferred not to be drawn into the controversy.

3. The indifferent, many of whom very possibly did not even read the petition.

4. Those who by reason of change of residence, absence, etc., did not receive the petition or received it so late that they thought it useless. The early copies of the petition were sent out just as many were starting for Denver and Los Angeles to the meetings of the American Surgical Association and the American Medical Association and a considerable number were starting for their summer holiday. While writing this footnote one of the June petitions has reached me, duly signed, making, therefore, 282 heard from and 202 unheard from.

How many belonged to each of these four classes I do not know.

May I add also the closing paragraph from General Leonard Wood's annual report, just issued: "It is the concensus of opinion in the Army that the canteen should be re-established. That opinion is concurred in by the undersigned."

This is the judgment of an army officer who is a physician as well, and one who also knows the sentiments of the Army.

The verdict, therefore, of the profession at large, seems to me, is very clear. Our petition referred only incidentally to the benefits of temperance, which we would approve, to the diminutions of discipline which certainly would be diminished by the restoration of the canteen. Our main plea was a purely surgical and humanitarian one, viz., the alarming increase of venereal diseases and their results to the soldier, his wife and children and to the community and the nation. On these points we speak "with authority and not as the scribes" and Pharisees.

To my mind the alternative is simply this: Re-establish the canteen in which simply beer may be sold, and sold under supervision of the military officers of each post; or, on the other hand, abolish the canteen and drive our soldiers to the vile saloons just outside the military reservations, resorts, which are brothels as well as saloons, where the soldiers are supplied with the vilest kinds of whiskey, not seldom drugged, robbed of their scant pay, and infected with either gonorrhoea or syphilis, which in turn they transmit to their wives and children, and it may be to others who are entirely innocent.

